# How can we improve stroke thrombolysis rates? A review of health system factors and approaches associated with thrombolysis administration rates in acute stroke care

**DOI:** 10.1186/s13012-016-0414-6

**Published:** 2016-04-08

**Authors:** Christine L. Paul, Annika Ryan, Shiho Rose, John R. Attia, Erin Kerr, Claudia Koller, Christopher R. Levi

**Affiliations:** 1 The University of Newcastle, University Drive, Callaghan, NSW 2308 Australia; 2 Hunter New England Health, Lookout Road, New Lambton Heights, NSW 2305 Australia; 3 Hunter Medical Research Institute, 1/Kookaburra Circuit, New Lambton Heights, NSW 2305 Australia

**Keywords:** Ischemic stroke, Thrombolysis, Implementation, Quality improvement, Health system change, Tissue plasminogen activator

## Abstract

**Background:**

Thrombolysis using intravenous (IV) tissue plasminogen activator (tPA) is one of few evidence-based acute stroke treatments, yet achieving high rates of IV tPA delivery has been problematic. The 4.5-h treatment window, the complexity of determining eligibility criteria and the availability of expertise and required resources may impact on treatment rates, with barriers encountered at the levels of the individual clinician, the social context and the health system itself. The review aimed to describe health system factors associated with higher rates of IV tPA administration for ischemic stroke and to identify whether system-focussed interventions increased tPA rates for ischemic stroke.

**Methods:**

Published original English-language research from four electronic databases spanning 1997–2014 was examined. Observational studies of the association between health system factors and tPA rates were described separately from studies of system-focussed intervention strategies aiming to increase tPA rates. Where study outcomes were sufficiently similar, a pooled meta-analysis of outcomes was conducted.

**Results:**

Forty-one articles met the inclusion criteria: 7 were methodologically rigorous interventions that met the Cochrane Collaboration Evidence for Practice and Organization of Care (EPOC) study design guidelines and 34 described observed associations between health system factors and rates of IV tPA. System-related factors generally associated with higher IV tPA rates were as follows: urban location, centralised or hub and spoke models, treatment by a neurologist/stroke nurse, in a neurology department/stroke unit or teaching hospital, being admitted by ambulance or mobile team and stroke-specific protocols. Results of the intervention studies suggest that telemedicine approaches did not consistently increase IV tPA rates. Quality improvement strategies appear able to provide modest increases in stroke thrombolysis (pooled odds ratio = 2.1, *p* = 0.05).

**Conclusions:**

In order to improve IV tPA rates in acute stroke care, specific health system factors need to be targeted. Multi-component quality improvement approaches can improve IV tPA rates for stroke, although more thoughtfully designed and well-reported trials are required to safely increase rates of IV tPA to eligible stroke patients.

## Background

Stroke causes five million deaths worldwide [1, 2] with escalating costs to the health system [3–6]. Most stroke cases (89 %) are admitted to hospital [7], with approximately 50 % of sufferers left deceased or dependent [8]. Thrombolysis using intravenous (IV) tissue plasminogen activator (tPA) is one of the few evidence-based acute stroke treatments [9, 10].

Despite the potential benefit offered by routine delivery of thrombolysis to eligible stroke patients, achieving and sustaining high rates of IV tPA delivery has been problematic.

While seeking treatment late is a major limiting factor on tPA delivery [11, 12], health system factors (i.e. circumstances that are determined by the health organisation or the health care provider rather than the individual) are important in improving access to thrombolysis for stroke patients. While there is no agreed benchmark for rates or levels of thrombolysis in practice, substantial change has been shown to be achievable such as an increase in tPA administration rate from 4.7 to 21.4 % of all stroke patients [13].

The narrow treatment window of 4.5 h from stroke onset, negative impacts of inappropriate treatment, along with the multi-step, multi-disciplinary testing, and decision-making process needed to determine thrombolysis eligibility would indicate that complex interventions are required to change thrombolysis rates [14]. Complex interventions are generally defined as those which involve a number of interacting components, require a number of behaviours or difficult behaviours, involve a number of groups or organisational levels and have a number of outcomes [14], each of which is directly relevant to thrombolysis for acute stroke. Barriers to treatment include delays in stroke recognition by staff [15], delays in obtaining and interpreting radiology imaging [16], inefficiencies in emergency stroke care and delays in obtaining treatment consent [17].

Study of the diffusion of new technologies indicates that while some innovations are largely adopted in less than 5 years [18], others may fail to become commonplace due to barriers or failures at a higher level [19]. In these contexts, the use of theoretical frameworks such as the Behaviour Change Wheel (BCW) [20] can be helpful to clarify the range of factors which may need to be addressed in order to effect change. The BCW describes the three essential conditions for behaviour change to occur: capability, opportunity and motivation; nine intervention functions and seven policy categories are required for whole system change [20]. Models and frameworks such as the BCW emphasise the importance of intervening not only at the level of the individual but also at an organisational or system level and at the broader policy level. While policy-level factors such as financial incentives may impact on thrombolysis over the long term [21], in the short to medium term, health service providers may have the greatest potential impact by acting at a health system or organisational level.

A number of cross-sectional studies have described associations between higher stroke tPA rates and system-level factors such as hospital size and hospital type [22, 23] or characteristics such as staffing [24] or stroke certification [25–28]. System-level approaches have been recommended to improve access to IV tPA and increase the proportion of patients receiving the treatment, including telemedicine and centralised hub and spoke models [29–31]. Some studies have described successful attempts to apply hospital pre-notification systems [13] or quality-improvement approaches (e.g. analysing performance, with systematic efforts to improve it, ultimately resulting in better health outcomes) [32, 33], to increase tPA implementation for stroke.

However, system changes require substantial resources and engagement with quality improvement programmes. To our knowledge, there are no published reviews of a broad range of evidence-based health system factors associated with increased IV tPA administration rates for stroke.

### Aims

The aim of this study is to identify the following:

- Health system factors associated with higher rates of IV tPA administration for ischemic stroke
- The effectiveness of system-focussed intervention strategies, which meet Cochrane Collaboration Evidence for Practice and Organization of Care (EPOC) study design guidelines, in improving IV tPA rates for treatment of ischemic stroke

## Methods

### Search strategy

The literature review in MEDLINE, CINAHL, EMBASE and PsycINFO spanned from January 1997 to May 2014 and was performed as title, abstract and full-text review by three independent reviewers, with ambiguous articles discussed as a group to reach agreement. The search period was selected to align with the 1996 approval of the “clot-buster” drug [34] and the release of the first tPA stroke guidelines [35]. Search terms were confirmed in consultation with clinical stakeholders and a medical librarian. Available MeSH headings were used; otherwise, a “title” field search was conducted.

Limitations included published original research, English language, humans, adults, and used a combination of keyword searches of “tpa.m_titl” OR “rtpa.m_titl” OR “Tissue Plasminogen Activator OR Tissue Plasminogen Activator.m_titl” OR “Fibrinolytic Agents OR Fibrinolytic Agents.m_titl” OR “Recombinant Proteins OR Recombinant Proteins.m_titl” OR “Thrombolytic Therapy OR Thrombolytic Therapy.m_titl” AND “Stroke OR Stroke.m_title” OR “Brain Ischemia OR Brain Ischemia.m_titl” OR “Cerebral Hemorrhage or Cerebral Hemorrhage.m_titl”.

### Inclusion criteria

The inclusion criteria are as follows:

- Studies that quantitatively assessed modifiable *health system factors* influencing rates of IV tPA for stroke; or
- *Intervention studies* aiming to improve rates of IV tPA administration for stroke

### Exclusion criteria

The exclusion criteria are as follows:

- Solely addressing patient characteristics such as age, race, education, income or clinical eligibility for thrombolysis
- No denominator for calculating tPA rates or not reporting a tPA rate
- Solely assessing intra-arterial tPA
- Addressing only community-directed or patient-directed activities or changes
- Hypothetical studies

### Data extraction

#### Health system factors

Using an extraction template, the following health system factors were extracted: sample characteristics, sample size, response rate, descriptors of setting, data collection method, rate and proportion of IV tPA administration, system factors addressed in relation to tPA delivery, factors affecting IV tPA rates, tPA criteria/guidelines and tPA time window. Using existing frameworks such as the BCW to categorise the identified health system factors was not successful as a number of the strategies could be categorised as having multiple intervention functions. Only three of the nine intervention functions and two of the seven policy functions described in the BCW were identified in the review. Therefore, a consensus process was used among the authors to identify practice-relevant categories under which to present the observational studies.

#### Interventions

Intervention studies were reviewed and categorised according to whether or not they met criteria for any of the four experimental designs defined and recommended by the EPOC design criteria. Data extracted were as follows: study design; setting; target group; study duration; intervention allocation; unit of analysis; allocation concealment; blinding; eligibility criteria; sample size; representativeness of sample; intervention conditions; outcome measures; statistical analysis; and findings.

### Quality control

Quality control involved second coding of a random sample of articles (10 %) at each review stage, i.e. initial extraction of studies and exclusion of ineligible studies (AR, SR, CP). Extracted data from all included studies were double-coded in full (AR, SR, CP, CK, JA, EK) and checked for agreement (AR). Agreement rates exceeded 90 % at all stages. All remaining differences in inclusions, exclusions and extracted data were discussed according to documented principles until consensus was reached, with subsequent re-coding completed wherever necessary.

### Analysis

For the experimental studies, synthesis of the data involved meta-analysis where possible. Only studies which had pre- and post-test data specifying the rate of thrombolysis for intervention versus control groups were included in the meta-analysis. For the four study outcomes that were sufficiently similar, a pooled meta-analysis of outcomes was conducted using StatsDirect (version 2.7.9., Cheshire, UK). Heterogeneity was checked using *I* ^2^ and if high, random effects (DerSimonian-Laird method) pooling was used. Narrative synthesis was used to describe outcomes for the remainder of the experimental studies which could not be included in the meta-analysis due to heterogeneity of outcomes. Narrative synthesis [36] involved verbal descriptions of the extracted data. For the observational studies, data synthesis involved tabulation of whether the study found a significant association with thrombolysis rate for any review-relevant factor followed by comparative narrative synthesis.

## Results

**Fig. 1:**
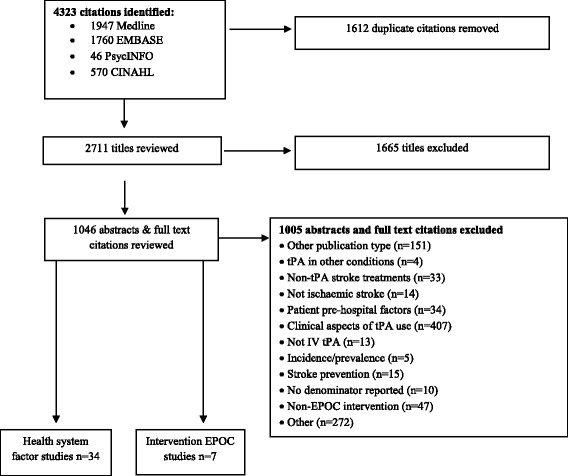
Inclusion and exclusion of citations

The search resulted in 4323 citations (MEDLINE *n* = 1947, EMBASE *n* = 1760, PsycINFO *n* = 46, CINAHL *n* = 570). As indicated in Fig. 1, 34 studies reported associations between health system factors and IV tPA rates for ischemic stroke. Seven intervention studies that reported an improvement in IV tPA rates met the EPOC design criteria. Forty-seven intervention studies were excluded as being either pre-test-post-test designs with no control group or pilot tests with post-test only data. The types of intervention strategies studied in the 47 excluded publications included the following: the introduction of stroke units or “code-stroke” protocols; support for regional sites (e.g. hub and spoke models, telemedicine); changes in hospital protocols, staffing or rostering; and the “Get With The Guidelines” programme [37].

### Health system factors

Table 1 summarises the 34 studies exploring associations between thrombolysis rates and health system factors. The majority (*n* = 19) of studies conducted multivariable analyses including both health system and patient factors. Health system factors were categorised post hoc and those with predominantly positive associations with tPA rates were as follows:

**Table 1:** Health system factors associated and not associated with higher thrombolysis rates

Health system factors	Studies finding *no* association with higher thrombolysis rate	Studies finding a *significant association* with higher thrombolysis rate
Travel time and location (environmental restructuring)^a^		
Shorter transport time or distance to hospital	[48–51]	[52, 53]
Urban (vs rural)	–	[54–56]
Centralised (hub model)	–	[57]
Training, skills and expertise (training and education)^a^		
Treated by a neurologist	–	[49, 56], [58] (no statistical test)
Admitted to or treated in a neurology department or stroke unit	[59]	[60, 61]
Academic/teaching hospital	[56]	[55, 60, 62–64], [65]^b^
Continuing medical education/formal stroke training	[33, 62]	[25]
Higher volume of stroke admissions/number of neuro beds	[56, 59]	[49, 61, 66]
Accreditation as medical centre	–	[49]
Facilities and staffing (service provision)^a^		
Emergency medical service or emergency department	[33]	[25]
Neurologists, stroke nurse, stroke unit or team	[33]	[25, 61, 62, 67]
Neurological/neuroimaging services	[62]	[25, 68]
Laboratory services	[25, 62]	–
Larger/higher volume hospital	[56, 61]	[69]
Arrival during “on” hours	[57, 70]	–
Arrival on weekend	[70]	[49, 71]
24 h or rapid CT/MRI	[62]	–
Intensive care unit (cat 1)	[72]	–
Stroke allocated beds	[33]	–
Organisational elements (guidelines and regulations)^a^		
Commitment of medical organisation or stroke centre director	[25]	[62]
Quality improvement outcomes or activities	[25, 62]	–
Pre-hospital notifications or triage tool	[73, 74]	[75]
Stroke-related certification	[76]	[77]
Ambulance agreements/protocols or training	[33]	[33] (borderline positive association)
Who interprets CT	[33]	–
Stroke-specific protocols	[62] (acute stroke protocol)	[25, 33, 62]
Transfer by a mobile emergency team or ambulance	–	[48, 50, 78, 79]

^a^Terms in parentheses refer to BCW intervention functions and policy categories

^b^Significant in univariate analysis only

- *Travel time and location*: e.g. urban rather than rural location, or a centralised/“hub” model linking outlying centres with other, generally larger, centres (environmental restructuring)
- *Training, skills and expertise*: treatment by neurologist or in a neurology department; admission to a stroke unit; treatment at academic/teaching hospital; treatment at a hospital with higher volume of stroke admissions or neurology beds; or accreditation as a “medical centre” (training and education)
- *Facilities and staffing*: having a neurologist, stroke nurse or stroke team; neurological or neuroimaging services; and weekend arrival (service provision)
- *Organisational elements*: use of stroke-specific protocols or transfer by ambulance/mobile emergency team rather than other means (guidelines and regulation)

The terms in parentheses refer to BCW intervention functions and policy categories.

### Effectiveness of system-focussed interventions

Two intervention studies [38, 39] compared telemedicine with a telephone-only approach under the “hub and spoke” model. This group were too diverse in methodology and measurement to be included in a pooled analysis. Therefore, a narrative outcome description is provided. Neither of the telemedicine studies found a significant difference in IV tPA rates or patient outcomes, with one [38] aiming to assess feasibility rather than effectiveness resulting in limited power to find any effect. Meyer et al. [39] identified significantly higher rates of correct treatment decisions in telemedicine-treated patients compared to the telephone-only group. A third study [40] explored a hub and spoke tele-consultation approach for one group of sites while a control group of sites proceeded with usual care. All sites found significant increases in IV tPA, while only tele-consultation sites significantly reduced mortality.

Four studies [32, 41–43] explored approaches using quality improvement methods. Of these, two [41, 42] found a significant effect on IV tPA rates and patient outcomes based on modified Rankin scores. Scott et al. [43] found a significant effect on IV tPA rates for some analyses, with no significant effect on service delivery measures or patient outcome. Schwamm et al. [32] reported that involvement in the Get With The Guidelines Stroke programme was associated with an improvement over time in thrombolysis rates for patients arriving within 2 h of symptom onset.

The four quality improvement studies were included in pooled analysis of tPA rates. As the heterogeneity of the studies was high (*I* ^2^ = 98 % [95 % CI = 97.1 to 98.5 %]), a random-effects model was used, and the pooled estimate should be treated with caution. A borderline significant effect was found, with a pooled odds ratio of 2.1 (95 % CI = 1.0 to 4.5) and; *X* ^2^ = 3.783689, *df* = 1, *p* = 0.05. The seven intervention studies are described in Table 2.

**Table 2:** Intervention studies meeting EPOC criteria for study design (*n* = 7)

Citation, trial name, design, setting	Target group, study duration	Randomization methods	Eligibility	Sample size, response rate, representativeness	Intervention conditions	Outcome measures	Statistical analysis	Findings
Demaerschalk 2010 [38], USA STRokE DOC AZ RCT Regional (spoke) and Academic Metropolitan (hub) hospitals	Hospital staff Dec. 2007–Oct. 2008	Unit of analysis: patient Concealed allocation: yes Blinded: no Allocation to condition: permuted block randomization of patients stratified by site	Patient: >18 years tPA window: onset <3 h.	Patient: *n* = 54 Hospital: *n* = 3 Response rate, 68.4 %. Representativeness: no demographic differences between groups Myocardial infarction higher in int. group (*p* < 0.02).	Int-1: audio and video contact with a certified stroke team at a hub site, who had access to medical history, performed NIHSS, and reviewed test results and CT images Int-2: a hub stroke consultant queried history, physical exam (including NIHSS), test results, CT report	tPA rate: denominator = acute stroke with <3 h onset. Service delivery: 1. Evaluation times (e.g. door-ED) 2. Correct treatment decision Patient outcomes: 1. Barthel Index (score 95–100) 2. mRS (score ≤2).	Cochran-Mantel-Haenszel test: comparison of correct decision rate between groups Fisher’s exact test: rate of tPA, rate of intracranial haemorrhage, mortality, 90 day mRS Wilcoxon rank sum test: 90-day Barthel Index and time comparisons	tPA rate: *Int-1*, 30 %; *Int-2*, 30 % Service delivery: 1. NS 2. NS Patient outcome: 1. NS 2. NS Note: insufficient power to assess difference in tPA rates between groups.
Dirks, 2011 [41], The Netherlands. PRACTISE Cluster RCT Hospitals	Hospital staff, including stroke neurologist and stroke nurse May 2005–Jan. 2008	Unit of analysis: hospital Concealed allocation: no Blinded: no Allocation to condition: hospitals randomised after pairwise matching on hospital type, tPA rate, stroke patients/year	Patient: >18 years Hospital: 100–500 stroke admissions/year tPA window: <4 h of onset	Patient: *n* = 1657. Hospital: *n* = 12. Response rate: Not reported. Representativeness: *patients:* mean age, sex distribution and mean NIHSS at admission were similar between groups	Int: 5 × half day (across 2 years) meetings based on Breakthrough Series model. Teams of stroke neurologist and stroke nurse were created, who noted barriers to tPA use, set goals and plan actions C: usual practices.	tPA rate: denominator = ischemic stroke, <4 h onset Service delivery: 1. Onset-to-door time (min) 2. Door-to-needle time (min) Patient outcome: 1. mRS <3 (at 3 months) 2. Quality of life—EuroQoL (at 3 months) 3. Mortality	Intention to treat Multilevel logistic and linear regressions: comparison of tPA use, mRS, QoL and mortality between intervention groups. Service delivery time analysis was adjusted for size, type and previous tPA rates, age, sex.	tPA rate: *Int*, 44 %; *C*, 39 % (unadjusted OR = 1.24 [1.02-1.51]). Service delivery: 1. NS 2. NS Patient outcome: 1. Poorer in C group 2. NS 3. NS
Meyer 2008 [39], USA STRokE DOC RCT Remote “spoke” hospitals	Hospital staff Jan. 2004–Aug. 2007	Unit of analysis: patient Concealed allocation: no Blinded: no Allocation to condition: patients randomised within permuted blocks stratified by site	Patient: >18 years and ability to sign consent tPA window: <3 h for treatment, but no time limit on eligibility for trial	Patient: *n* = 222 (111 vs 111) Hospital: *n* = 4 Response rate: *Patients:* Not reported. Representativeness: No demographic differences between groups. Int-1 had higher NIHSS score at presentation than Int-2 (*p* < 0.005).	Int-1: telemedicine (including video) consultation with patient by hub consultant including CT imaging Int-2: telephone consultations for spoke sites with hub consultants Hub provided treatment recommendations for both groups	tPA rate: denominator = acute stroke. Service delivery: 1. Correct treatment decisions 2. Stroke onset to each point of care pathway (min) Patient outcome: 1. Barthel Index (score 95–100). 2. mRS (score ≤2).	Fisher’s exact test: difference in tPA rate, functional outcomes	tPA rate: *Int-1,* 28 %; *Int-2*, 23 % (OR = 1.3 [0.7–2.5], *p* = NS). Service delivery: 1. Greater in Int-1 compared to Int-2 (98 vs 82 %, OR = 10.9 [2.7–44.6], *p* < 0.001). 2. Few differences in service delivery times. Patient outcome: 1. No difference between groups 2. No difference between groups
Morgenstern et al. 2003 [42], USA TTL Temple Foundation Stroke Project CBA Hospitals in two communities	Community members and hospital staff Feb. 1998–Sept. 2000	Unit of analysis: patient Concealed allocation: no Blinded: no Allocation to condition: comparison community selected to match chosen intervention community	Patient: >21 years and county resident tPA window:<3 h	Patient: *Phase 1: n* = 277 (136 vs 141) *Phase 2: n* = 499 (266 vs 233) *Phase 3*: *n* = 150 (80 vs 70) Hospital: *n* = 10 Response rate: *Patients:* N/A *Hospitals:* not reported Representativeness: hospital characteristics reported	Int: community mass media, hospital-based systems change via multi-disciplinary team development of ED protocols, problem solving, medical education, feedback. C: not specified.	tPA rate: denominator = ischemic stroke Service delivery: 1. Delay time to hospital 2. Staff-reported barriers to treatment Patient outcome: none assessed	Fisher’s exact test: rate of tPA ANOVA: delay in times	tPA rate: *Int (phases 1–3)*: 2.2, 8.6, 11.2 % (*p* < 0.007); *C (phases 1–3):* 0.7, 0.9 %, (*p* = NS) Service delivery: 1. No difference in either group 2. Reduction for Int group only (no statistical test)
Schwamm et al. 2009 [32], USA ITS Academic and community hospitals	Hospitals April 2003-July 2007	Unit of analysis: hospital Concealed allocation: N/A Blinded: N/A Allocation to condition: N/A (ITS design)	Patient: Principal diagnosis of stroke or TIA, arrival <2 h from onset, ICD-9. Retrospective chart review to confirm stroke/TIA Hospital: >30 patients	Patient: *n* = 322,847 (ischemic = 73.2 %; TIA = 26.8 %) Hospital: *n* = 790 Response rate: Unclear. Staggered recruitment over 4 years. By Jan. 2007, 8.35 % hospitals had dropped out (*n* = 66) Representativeness: hospital characteristics provided	Int: quality improvement (Get With The Guidelines [GWTG]) programme, with organisational meetings, tool kits, collaborative workshops, hospital recognition, decision support information, performance feedback.	tPA rate: denominator = stroke or TIA, and arrival <2 h of onset Service delivery: none assessed Patient outcome: 1. Symptomatic intracranial haemorrhage within 36 h of tPA	Cochran-Mantel-Haenszel test: mean score for changes in rate of tPA and intracranial haemorrhage over time	tPA rate: significant increase from baseline (42.1 %) to year 5 (72.8 %; *p* < 0.0001). Patient outcome: 1. NS over time Greatest improvement (composite performance/program year in GWTG) in hospitals with more beds (*p* < 0.0001), larger annual stroke volume (*p* < 0.0001) and teaching status (*p* < 0.0001)
Scott et al. 2013 [43], USA INSTINCT Cluster RCT Community hospitals	Physicians, pharmacists, nurses, EMS, admin teams Jan.–Dec. 2007	Unit of analysis: hospital Concealed allocation: no Blinded: no Allocation to condition: within pairs, hospitals were randomised to intervention or control groups. Randomisation reversed for three pairs to achieve greater urban/rural balance	Hospitals: discharging ≥100 stroke patient/year, <100 000 ED visits/year and non-academic stroke centres tPA window: not specified	Hospitals: *n* = 24 Response rate: 83 % Representativeness: not reported	Int: clinical practice guideline promotion, development of local stroke champions, continuing education, telephone support for treatment decision, academic detailing, audit and feedback C: usual practices	tPA rate: denominator = ischemic stroke Service delivery: 1. Adherence to tPA guidelines Patient outcome: 1. Safety data from proportion of patients (2.2 %), with reported haemorrhage	Intention-to-treat (ITT) and target population (without one pair that was excluded after randomisation) Generalised linear mixed model: assumed intra-hospital correlation between tPA rates at pre- and post- intervention periods	tPA rate: *ITT:* Int (pre and post), 1.25 and 2.79 %; C (*n* = 1; pre and post), 1.25 and 2.10 %. Int vs C, *p* = NS. *Target analysis:* Int (pre and post), 1.0 and 2.62 %; C (pre and post),1.09 and 1.72 %. Int vs C, RR = 1.68 [1.09–2.57], *p* = 0.02 Service delivery: 1. NS difference between groups Patient outcome: 1. NS difference between groups
Theiss et al. 2013 [40], Germany CBA Comprehensive stroke centres, and primary care hospitals	Hospitals 2006–2009	Unit of analysis: hospital Concealed allocation: no Blinded: not reported Allocation to condition: hospitals matched on beds, distance from closest hub site and departments of internal medicine	Hospitals: not reported No study hospitals had specialised stroke care prior to study start	Hospitals: *n* = 15 Response rate: not reported. Representativeness: not reported	Int: tele-consultation service. Consisted of hub (*n* = 5) and spoke (*n* = 5) sites C: usual practices	tPA rate denominator: all stroke Service delivery: none assessed Patient outcome: 1. Intracerebral haemorrhage 2. Mortality	Mean and SEM: for descriptive data Student *t* and Fisher exact tests: longitudinal and pairwise comparisons, pooled ischemic stroke mortality	tPA rate: *Hub sites*: (pooled) increased 4.2 to 7.7 % (*p* < 0.0001); *Spoke sites:* (pooled) increased 1.1 to 5.9 % (*p* < 0.0001); *C:* (one hospital only) increased 0.8 to 5.7 % (*p* = 0 . 03). Patient outcome: 1. NS 2. Significant decreases in spoke site only (10.3 to 7.3 %, *p* = 0.03)

*Abbreviations: C* control group, *CBA* controlled before and after trial, *CT* computer tomography, *ED* emergency department, *EMS* emergency medical service, *RCT* randomised controlled trial, *Int* intervention group, *ITS* interrupted time series, *mRS* modified Rankin score, *NIHSS* National institute of Health Stroke Scale, *TIA* transient ischemic attack, *tPA* tissue plasminogen activator, *QoL* quality of life, *N/A* not applicable

## Discussion

This systematic review brings together the empirical evidence regarding potential strategies for improving thrombolysis rates for acute stroke. The review data provide a basis on which stroke service providers can identify which strategies are more likely to be good investments for increasing rates of thrombolysis. As per the literature regarding complex interventions [14] and frameworks such as the BCW [20], a range of strategies or factors are related to achieving change in thrombolysis rates. Of note is that the literature only addresses three of the nine intervention functions and two of the seven policy categories raised in the BCW framework, suggesting a much wider range of strategies could be tested in the future.

A small number of system-related factors are associated with higher rates of IV tPA administration for ischemic stroke. Systems-change interventions, based on multi-component quality improvement approaches, can increase the proportion of eligible stroke patients receiving IV tPA.

The observational literature regarding factors associated with higher stroke tPA rates was heterogeneous in methodology and types of factors assessed, but it is unclear whether each study had sufficient power to detect an association for each factor. The literature indicates that health systems should aim to ensure that most stroke patients are treated in a way that minimises access disadvantages for rural populations; maximises access to neurological and stroke-specific expertise and experience; ensures stroke units are widely available; and implements stroke-specific protocols. The association of higher IV tPA rates with treatment at a teaching hospital or a hospital with larger stroke or IV tPA treatment volume suggests that expertise and experience within such settings is key to increased IV tPA rates. The mixed findings regarding the importance of treatment at a larger hospital and arrival during “on” hours or weekends indicate that greater size and availability of staff alone do not produce higher IV tPA rates. However, it must be noted that observational studies cannot be used to draw definitive conclusions regarding causation. The observational studies were also largely retrospective in design and had limited capacity to identify and assess confounding factors. Therefore, a greater focus must be directed towards the data from the experimental or intervention studies.

Organisational elements such as stroke certification and quality improvement activities were not associated with higher IV tPA rates. One study [25] failed to find an association between facilities, staffing and organisational elements and quality improvement outcomes or activities. These elements are often the focus of system-change interventions and can be resource intensive to implement. Therefore, robust experimental studies are essential to providing clarity about cost-effective approaches to improved IV tPA rates. Organisational elements such as stroke-related certification or time on the Get With The Guidelines programme did not increase IV tPA rates.

The intervention studies suggest that while quality-improvement or system-change interventions can be effective in increasing IV tPA rates, studies are heterogeneous and effects may be small or inconsistent. The PRACTISE trial [41] found a positive effect on IV tPA rates and patient functioning following a Breakthrough Series intervention. The INSTINCT trial [43] reported a positive effect only when the analysis focussed on a subset of study sites. The INSTINCT intervention placed less emphasis on collaborative meetings compared to the PRACTISE trial but included stroke champions, education/support for treatment decision making and performance feedback [43]. The Morgenstern et al. study [42] identified a greater increase in IV tPA rates in intervention sites, compared to control sites.

This study [42] differed from the two other studies by including community-focussed mass media. It also included hospital-based change via multi-disciplinary teams, development of emergency department protocols, problem solving, medical education and performance feedback. Given the small number of hospitals involved, the choice of patient rather than hospital as the unit of analysis, and the lack of any head-to-head analysis across groups, some caution should be applied to interpreting the results of the Morgenstern et al. study [42].

Other studies support the finding that quality improvement strategies can provide modest positive effects on other aspects of stroke care [32, 44, 45]. The “Stroke 90:10” trial found an 11 % relative improvement in some aspects of initial assessment and care for stroke patients following collaborative quality improvement [45]. Another study involving workshops, education, site-based teams, performance feedback and decisional support suggests that improvements in thrombolysis occurred over time [32]. While the study could be classified as an interrupted time series based on quarterly measurements over 4 years, the analysis did not follow usual approaches to analysing time-series data. Although the cost of IV tPA administration was not addressed in the reviewed studies, the scope of multi-component, multi-site interventions suggests the resources required are substantial.

Two studies [38, 39] compared telemedicine with a telephone-only approach, focusing on environmental restructuring rather than quality improvement to increase IV tPA delivery. Conclusions are difficult to make, as neither study indicated sufficient power to detect a difference in IV tPA rates. The study of tele-consultation compared to usual care [40] suggested, but did not conclusively demonstrate, patient outcome benefits, given a failure to statistically examine experimental versus control site outcomes. A later pooled analysis confirmed telemedicine consultations were not associated with increased thrombolysis rates [46]. While these changes may increase access to expert care, they lack robust evidence.

Observational and intervention data suggest that optimising IV tPA administration requires availability of expertise and protocols. Intervention studies suggest more in-depth reporting of the degree to which various intervention strategies may assist in understanding the best way forward. While multi-component approaches appear promising, two important questions emerge:

- Could a comprehensive intervention approach, encompassing the range of strategies represented in the reviewed studies, achieve a more substantial increase in IV tPA rates than that found to date? If so, what is the cost-benefit?
- Could a more streamlined quality improvement approach be identified, using a subset of elements? This may require comprehensive and systematic approaches to study the implementation of prior and future multi-component interventions, followed by trials using a subset of “best-bet” strategies.

The broader context is also important to consider, such as the financial incentives to hospitals for or against thrombolysis delivery in certain settings [21]. Adapting system thinking where components of the health system are dynamic and interlinked may assist in further understanding the network of relations and feedback loops impacting on the uptake of new innovations [47]. It may also be useful to develop a broader theoretical framework that could be applied to future studies in this area.

Limitations should be considered when interpreting the study tables: firstly, the reported rates of IV tPA (see Table 2) are dependent on denominator and eligibility criteria, which can affect the power of the study to detect a difference in the outcome; secondly, the variability in factors explored across studies of descriptive health system factors limits the ability to make comparisons among studies; and finally, the nature of changing care at a system level limits design rigour such as the ability to blind sites to group allocation. As a “Google” search was not used, a small number of unpublished studies may not have been identified.

## Conclusion

Access to teaching hospitals and hospitals with larger stroke and IV tPA treatment volumes is associated with increased IV tPA administration rates for stroke, although results should be viewed against variability in eligibility criteria and type of denominator used. Interventions aiming to increase rates of IV tPA are resource intensive and comparisons between studies are difficult due to insufficient power and limitations in study analysis. More empirical data regarding the effects of efforts to improve access to thrombolysis for those living long distances (e.g. mobile thrombolysis) from a tPA-capable hospital and 24-h availability of expertise in acute stroke care are required, as is more thoughtfully designed and well-reported trials of quality improvement interventions.
